# Seroepidemiological investigation of COVID-19: A cross-sectional study in Jundiai, São Paulo, Brazil

**DOI:** 10.1371/journal.pgph.0000460

**Published:** 2022-09-09

**Authors:** Marília Jesus Batista, Carolina Matteussi Lino, Carla Fabiana Tenani, Luciane Zanin, Andréa Tenório Correia da Silva, Monica Vannucci Nunes Lipay, Carolina de Lima Rossi, Jane Rodrigues Tonetti, Andréia Pinto de Souza, Fabiana Barrete de Alcântara Fredo, Evaldo Marchi

**Affiliations:** 1 Department of Community Health, Jundiai Medical School, Jundiaí, São Paulo, Brazil; 2 Department of Health Sciences and Child Dentistry, Piracicaba Dental School, University of Campinas (UNICAMP), Piracicaba, São Paulo, Brazil; 3 Jundiaí City Hall Prefeitura Municipal de Jundiaí, Jundiaí, São Paulo, Brazil; National University Singapore Saw Swee Hock School of Public Health, SINGAPORE

## Abstract

The dramatic increase in the number of COVID-19 cases has been a threat to global health and a challenge for health systems. Estimating the prevalence of infection in the population is essential to provide support for action planning. Within this scenario, the aim of the present study was to analyze the seroprevalence and associated factors of COVID-19 Jundiaí, São Paulo, Brazil. This cross-sectional study was conducted from June 1st to June 19th, 2020. The participants were patients with respiratory symptoms who sought Primary Care Units (UBS) (n = 1,181) and subjects recruited from randomly selected households by probability sampling (n = 3,065), as screening strategy. All participants, in both phases, were submitted to SARS-CoV-2 rapid antigen tests (IgG and IgM) and responded to a questionnaire including sociodemographic characteristics based on Behavioural Insights for COVID-19. Total seroprevalence (positive/negative) was the outcome and the independent variables were sociodemographic variables, health behavior and signs/symptoms. The chi-squared test was used for association analysis (p<0.05) and variables with p<0.20 were entered into the logistic regression model (p<0.05). A total of 1,181 subjects from the UBS and 3,065 from the selected households participated in the study. The seroprevalence was 30.8% in the UBS and 3.1% in the households. The adjusted logistic regression identified that lower educational level (OR 2.68; 95%CI 1.59–4.54), household member testing positive (OR 1.67; 95%CI 1.16–2.39), presence of anosmia (OR 3.68, 95%CI 2.56–5.28) and seeking UBS (OR 3.76; 95%CI 2.08–6.82) was risk factors to test positive for SARS-CoV-2. Estimating the seroprevalence in the population was important to know the disease extension that was higher than the notified cases. These results showed socioeconomic aspects associated with COVID-19 even adjusted by symptoms. Populational epidemiologic studies that investigate the associated factors of COVID-19 are relevant to plan strategies to control the pandemic.

## Introduction

In December 2019, a novel type of human coronavirus denominated SARS-CoV-2 was identified in Wuhan, China, which spread quickly around the globe and led the World Health Organization (WHO) to declare the disease caused by it–COVID-19 –a pandemic on March 11, 2020 [1–3]. In Brazil, the first case of infection of the COVID-19 was detected in February 2020; on 15 March 2022, 20,928,008 cases and 584,421 deaths had been reported in the country, with the southeastern region accounting for the largest number of cases, with 39.03% of cases and 47.75% of deaths [4]. At the beginning, the city of São Paulo—capital of the State of São Paulo—was the epicenter but SARS-CoV-2 virus spread rapidly to the interior municipalities [5] such as Jundiaí/SP, located 57 kilometers away from the city of São Paulo. The first two COVID-19 cases in the city of Jundiaí were confirmed in March 2020 and the first death in April 2020. Currently (15 March 2022), the municipality has more than 74,000 confirmed cases, 1,760 of whom had died [6]. Jundiai was in 12^th^ position in the COVID-19 ranking of the State of São Paulo, which has 645 municipalities, and is the 15^th^ in the population size.

The dramatic increase in the number of cases and deaths due to the SARS COV-2 has become a threat to global health and a challenge for health systems because of the demand for health professionals, intensive care beds, mechanical ventilators, and personal protective equipment for professionals. In addition, the increase in cases represents a challenge for surveillance and healthcare systems and how the disease is notified in the country [7–9].

International organizations like the WHO, the World Federation of Public Health Associations, and the Centers for Disease Control and Prevention of the United States have developed and disseminated measures to reduce the spread of COVID-19, including hand hygiene, the use of face masks and personal protective equipment, particularly by health professionals (N95 mask, face shield, and safety glasses), and social distancing [7,10–12]. Another WHO recommendation is the focus on screening, with testing and diagnosis of positive cases, in order to isolate infected individuals and to quarantine contacts [13].

Most of the studies that investigated the epidemiological profile of COVID-19 infection have focused on severe forms of the disease using data obtained from hospital admissions and notification forms [14,15]. In Brazil, a population study on the seroprevalence of COVID-19 carried out in three stages also investigated the asymptomatic population and individuals with subclinical symptoms and found a higher prevalence of the disease than that estimated by the number of cases reported at the beginning of the pandemic in the country [16]. Thus, epidemiological studies are relevant because the growing number of cases and deaths caused by COVID-19 have an impact in health systems and compromise their response capacity [16]. Population-based epidemiological studies are extremely important for estimating the prevalence of infection in the population and for identifying the frequency of cases with mild or subclinical symptoms and asymptomatic patients that did not seek a health service but could sustain the infection [13]. Seroepidemiological surveys using WHO standards are therefore of paramount importance to identify infected individuals and associated factors in order to support measures necessary for the treatment and definition of strategies for preventing and combating COVID-19 disease [17].

Within this context, the aim of the present study was to investigate the prevalence of COVID-19 seropositivity and the factors associated with it in the municipality of Jundiai, São Paulo, Brazil, before the introduction of the vaccine against COVID-19.

## Materials and methods

### Study location and design

**Fig 1:**
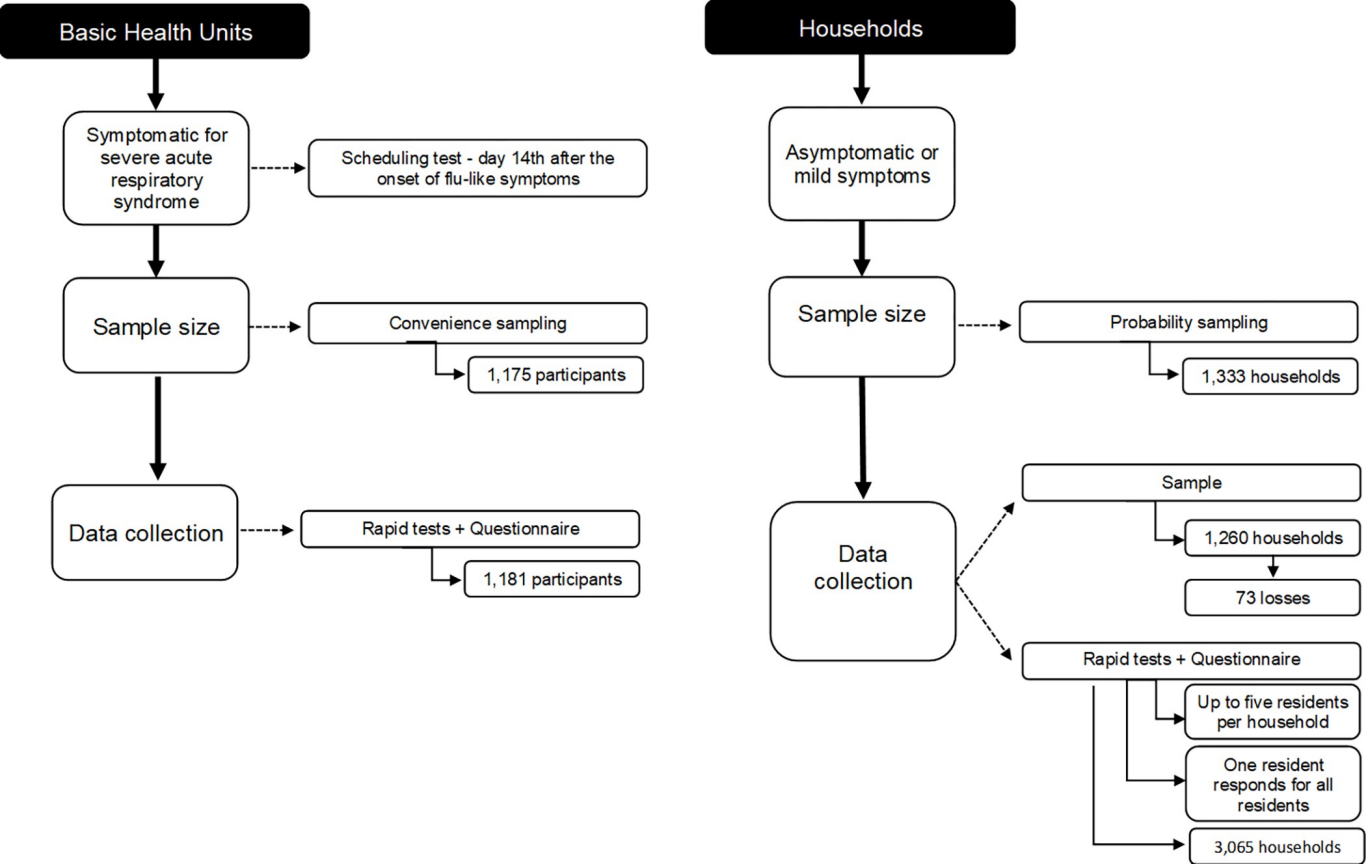
Data collection procedure in studies conducted at the basic health units and households, Jundiaí, São Paulo, 2020. The figure shows the steps of collection of the data obtained in the present study.

This is a cross-sectional study conducted in the municipality of Jundiaí, State of São Paulo, Brazil, from June 1^st^ to June 19^th^, 2020, as a cooperation between the Municipal Health Management and Promotion Department, and the School of Medicine of Jundiaí (FMJ). The data were collected simultaneously in two phases: at Primary Care Units (UBS in the Portuguese acronym) and at selected households (Fig 1).

Jundiai/SP is a municipality in the state of São Paulo, Brazil. The estimated population in 2019 was 403,769 inhabitants [18]. The municipality is part of the 42 cities that belong to the Regional Department of Health III–Campinas and it is divide into seven administrative regions (Center, East, Northeast, Northwest, North, West, and South) and four health regions (regional I, regional II, regional III and regional IV), with a total of 35 health units [19]. According to the Brazilian Institute of Geography and Statistics (IBGE), the 2010 Human Development Index of the municipality was 0.822 [20], a value considered to be very high compared other Brazilian municipalities.

### Sample size and sample selection

The estimated sample size for data collection at the UBS was 940 cases. This estimate was obtained assuming a prevalence of 10% among all symptomatic patients tested, an alpha of 0.05, and an odds ratio (OR) of 3.0, as well as a 95% confidence interval. Considering 20% of losses or dropouts, the final sample size was 1,175 participants.

For selection the sample, in this phase, it was calculated for each UBS a different number of participants, according to the weekly average number of rapid tests performed based in the period before the study (from April to May 2020) in order to considering the different size of the population the units attend. This data was obtained from the Health Secretary of the Municipality. The data collection at that unit was stopped when the number sample size was reached. All individuals that were scheduled at the health unit to carry out a rapid test (IgG and IgM for SARS-CoV-2) after 14 days the onset of flu-like symptoms were invited to participate of the study.

For the household phase, the estimated sample size was 1,067 households. This calculation assumed an estimated population of 400,000 inhabitants, an error margin of 3%, a prevalence of 50%, and an alpha of 5%. In this phase, 20% of losses or dropouts were also considered, resulting in a final sample of 1,333 households.

**Fig 2:**
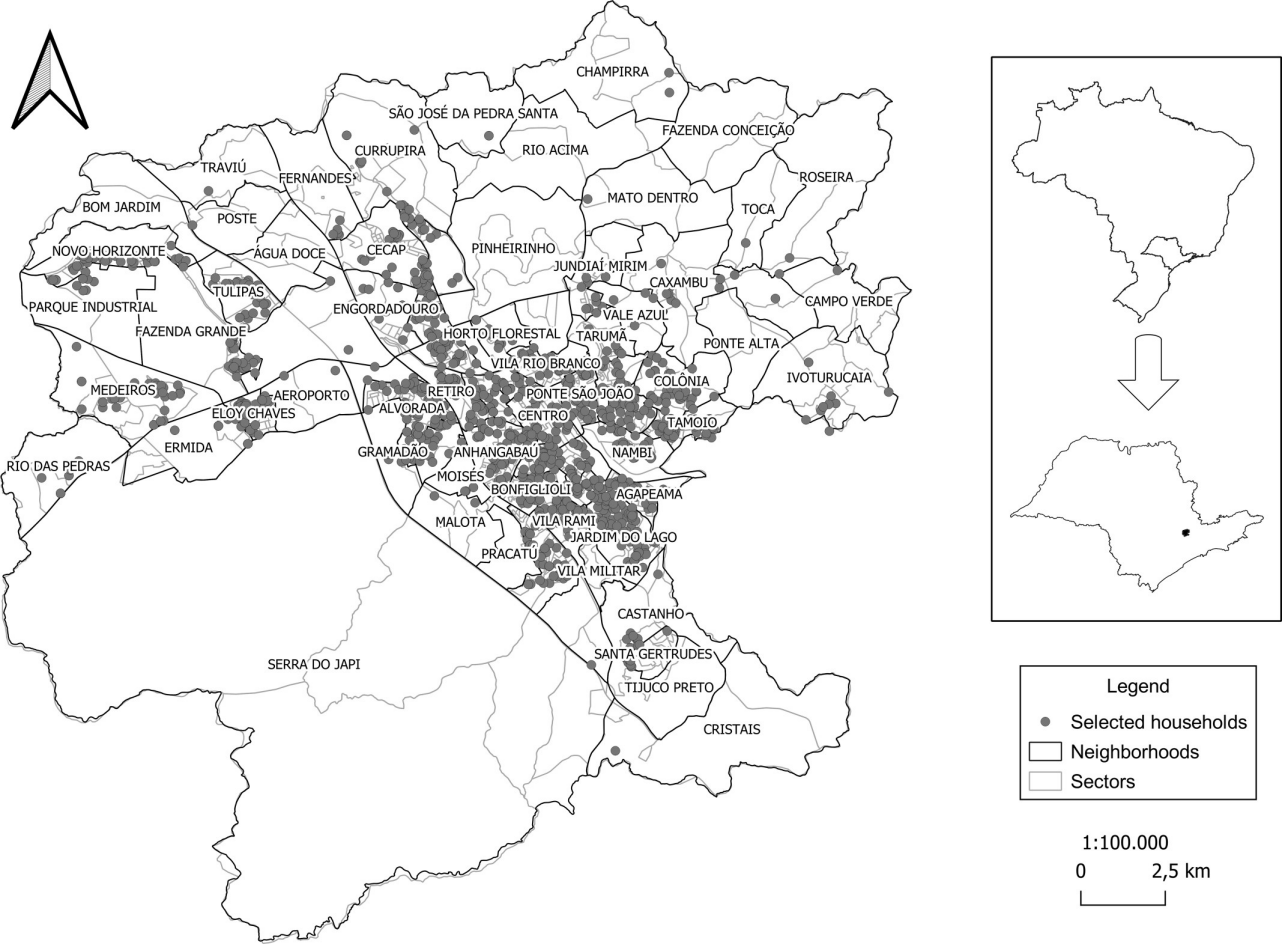
Households selected for data collection, Jundiaí, São Paulo, Brazil, 2020 (n = 1,333). The map shows the division of the municipality into neighborhoods and the distribution of the households randomly selected for the study. The base layer of the map is available in the Brazilian Institute of Geography and Statistics: https://www.ibge.gov.br/geociencias/organizacao-do-territorio/malhas-territoriais/15774-malhas.html?edicao=30138&t=downloads.

The participants were selected by probability sampling in which an updated list was first obtained from the Urban Property and Land Tax registration that contained all neighborhoods and their respective numbers of households. This list was used to calculate the initial sample size for each neighborhood–considering all neighborhoods in the municipality–with the probability being proportional to the number of households. Next, the households in each neighborhood that would compose the sample were randomly selected using Microsoft Excel (Fig 2). Due to the possibility of refusals and/or empty households, two substitutes were also drawn for each previously selected house.

Inclusion criteria for both phases (UBS and households) were individuals who were residents in Jundiaí, signing the free informed consent form, having more than 18 years old. Having no symptoms at moment of the study. The exclusion criteria were subjects under the influence of alcohol and/or toxic substances that prevented them from completing the questionnaire were excluded.

### Data collection

The health professionals were trained for application of the rapid tests. All members of the data collection teams participated of a training for the questionnaire and study design by the researchers during face-to-face-meetings and online, with a workload of 16 hours.

Data from UBS participants were collected at the health unit, when the patient scheduled at health unit to carry out a rapid test (14 days after the onset of flu symptoms). A person trained to applying the study’s questionnaire invited the patient to participate of the study and answering the questions, upon consenting to participate, and collected the result of the test (positive or negative).

Ten teams collected the data from the households. These teams consisted of a higher health professional (responsible for carrying out the rapid tests), a community health agent, and two medicine and/or nursing interns, with access to vehicles with sufficient space for organizing the material and carrying out the rapid tests.

In order to reach the sample size (n = 1,330), each team from the 10, received a spreadsheet with 133 households for invitation of the participants. Up to five rapid tests could be performed per household and one of the residents of legal age (more than 18 years old) was selected to answer the questionnaire. If there were more than five residents, the subjects to be tested were random selected by enumerating each resident. If they agreed to participate, elderly residents and individuals with risk comorbidities were automatically selected for the tests (because age and comorbidities are risk factors for severe COVID-19). If the result was positive, the participant was referred to a health service. In case of refusals and/or empty households, the team should go to the substitute-selected household, in the same location. All data on absences, substitutions and refusals were recorded to calculate the non-response rate.

In both phases–UBS and household–Vyttra Smart Test COVID-19 rapid tests were used for determination of the seroprevalence (sensitivity of 88.7% for IgM and 91.4% for IgG; specificity of 89.1% for IgM and 94.0% for IgG) [21]. These tests enable the qualitative and differential detection of SARS-CoV-2 IgM and IgG antibodies in serum, plasma or blood. The blood sample was obtained by fingerstick, and the test was performed according to manufacturer recommendation. In addition, the participants answered a questionnaire composed of 48 questions divided into five blocks: sociodemographic data (18 questions); symptoms, comorbidity and use of health services (13 questions); knowledge of the participant about the disease (5 questions); health behavior (9 questions) and the participant’s perceived impact of the pandemic (3 questions). The questionnaire was applied with the aid of a tablet. The instrument was based on the Behavioural Insights for COVID-19 tool recommended by the WHO [22]. It should be noted that all team members were properly dressed and the material for testing was stored in accordance with WHO recommendations [23].

### Variables

The dependent variable was the presence of a positive rapid test result for COVID-19 (IgG and/or IgM), considering adjustment for the test location (UBS or household) based on the different sampling strategies.

The following independent variables were considered: (i) sociodemographic (age [13–17, 18–39, 40–59, 60 years or more], sex [female, male], self-reported race/skin color [white, mixed-race, black, yellow], educational level [illiterate, elementary school, high school, higher education, postgraduation], income in minimum wages [up to 1, 1 to 2, 2 to 3, 3 to 4, 5 or more, no income]); (ii) behavioral (routine affected by the pandemic [routine remained the same or routine was affected by the pandemic, leaving home only for essential needs], adherence to preventive measures [adhered to the use of mask, hand sanitizer and social distancing, did not adhere, or household members did not adhere], adherence to social distance [adhered to social distancing or did not adhere] and report of positive cases among household members [yes or no]); (iii) signs and symptoms (presence of signs and symptoms at the time of the test [yes or no], type of sign and symptom [yes or no for each sign or symptom]: fever, cough, runny nose, headache, body aches, tiredness, diarrhea, sore throat, shortness of breath/dyspnea, anosmia, taste disorders, nail lesions, and skin alterations).

The categories 13 to 17 years (age), yellow skin (skin color), and illiterate (educational level) of the sociodemographic variable were excluded from the bivariate and logistic regression analyses because of the small number of participants in these categories.

### Statistical analysis

First, the data were submitted to descriptive analysis to calculate the frequency and percentage of responses. Next, bivariate analysis (Chi-square and Fisher’s exact test) was performed to compare the sample in the UBS and the households, and to evaluate the association between the outcome and the independent variables. For logistic regression, four blocks containing variables that showed p<0.20 in bivariate analysis were elaborated: 1) socioeconomic–age, income, skin color, and educational level; 2) test location–UBS or household; 3) behavior–routine affected by the pandemic, adherence to social isolation, and household member with a positive test; 4) symptoms–fever, tiredness, taste disorders, anosmia, shortness of breath/dyspnea, and sore throat. The final model was obtained by adjusting the blocks. The analyses were performed using SPSS 20.0 and a level of significance of 5% was adopted.

The study was approved by the Research Ethics Committee of the School of Medicine of Jundiaí on May 21, 2020, under number 4.040.674 (Ethical Clearance Certificate: 31748920.1.0000.5412). The data were collected after the participants had signed the free informed consent form (ICF).

## Results

The data collection at the UBS included 1,181 subjects with respiratory symptoms who sought the health units for a consultation and rapid test for COVID-19. The seroprevalence found was 30.8%. Among the diagnosed subjects who answered the questionnaire, there was a predominance of women (63.7%), subjects with self-reported white skin color (67.2%), and subjects aged 18 to 39 years (52.8%).

In the household data collection step, 1,260 households were included, totaling 3,065 tested participants. The seroprevalence evidenced at this stage was 3.1% among all subjects tested. In each of the participating households, one resident was selected to apply the characterization instrument, totaling 1251 respondents. There was a higher prevalence of asymptomatic participants (97.2%), women (64.3%) and subjects with self-reported white skin color (72.3%). Regarding age, most participants were between 40 and 59 years old (39.7%). The age group over 60 years accounted for 33.1% (Table 1).

**Table 1:** Sociodemographic characteristics of the participants in the basic health unit and household samples of the seroepidemiological survey, Jundiai, São Paulo, June 1^st^ to June 19^th^ 2020 (n = 2,432).

Variables	Household (n = 1,251)		UBS (n = 1,181)		p-value
	n	%	n	%	
Age (years)					
18 to 39	333	26.6	624	52.8	<0.001
40 to 59	497	39.7	441	37.3	
60 or more	414	33.1	116	9.8	
Unknown	4	0.3	0	0.0	
Sex					
Female	804	64.3	752	63.7	0.402
Male	447	35.7	429	36.3	
Skin color					
Black	69	5.5	73	6.2	<0.001
Mixed-race	244	19.5	296	25.1	
White	905	72.3	794	67.2	
Yellow	30	2.4	10	0.8	
Did not respond	3	0.2	8	0.7	
Educational level					
Illiterate	16	1.3	0	0.0	<0.001
Elementary school	292	23.3	146	12.4	
High school	446	35.7	562	47.6	
Higher education/Postgraduation	496	39.6	458	38.8	
Did not respond	1	0.1	15	1.3	
Income (in minimum wages**)					
Up to 1	82	6.6	56	4.7	0.188
1 to 2	197	15.7	214	18.1	
2 to 3	241	19.3	218	18.5	
3 to 4	227	18.1	243	20.6	
5 or more	355	28.4	337	28.5	
No income	11	0.9	13	1.1	
Did not respond	138	11.0	100	8.5	
Pandemic affected routine					
Did not leave the house	143	11.4	152	12.9	<0.001
Left the house only to go to work	273	21.8	566	47.9	
Left the house only for emergencies	798	63.8	424	35.9	
Did not change routine	37	3.0	39	3.3	
Adherence to preventive measures					
I did not adhere because I do not believe the measures to be effective/important	14	1.1	11	0.9	0.165
I did not adhere because I did not understand the measures	2	0.2	5	0.4	
I tried but I do not have the money to buy a mask/sanitizer	5	0.4	0	0.0	
Adhered to the measures but the household members did not	103	8.2	98	8.3	
Did not adhere but the household members did	65	5.2	75	6.4	
All family members adhered	1062	84.9	992	84.0	
Adherence to social distancing					
Yes, because I could not work at home	117	9.4	351	29.7	<0.001
Yes, because I had to work outside home to pay bills	42	3.4	32	2.7	
Yes, because people living with me did not adhere	27	2.2	45	3.8	
Yes, because I received visits from parents/friends	36	2.9	19	1.6	
I did not adhere because I do not believe the measure to be effective	17	1.4	16	1.4	
No, I only left home for emergencies	1011	80.8	718	60.8	
Household members testing positive					
No	1189	95.1	792	67.1	<0.001
Yes	61	4.9	389	32.9	
Presence of signs and/or symptoms at the time of the test					
Yes	35	2.8	416	33.3	<0.001
No	1216	97.2	765	61.2	
Type of sign and/or symptom					
Fever	3	0.2	79	6.7	0.089
Cough	15	1.2	216	18.3	0.196
Runny nose	14	1.1	101	8.6	0.036
Headache	4	0.3	168	14.2	<0.001
Body aches	9	0.7	131	11.1	0.307
Tiredness	3	0.2	95	8.0	0.032
Diarrhea	1	0.1	51	4.3	0.069
Sore throat	5	0.4	84	7.1	0.275
Shortness of breath/dyspnea	9	0.7	115	9.7	0.491
Anosmia	2	0.2	111	9.4	0.002
Taste disorders	4	0.3	106	9.0	0.042
Nail lesions	0	0.0	0	0.0	-
Skin alterations	1	0.1	13	1.1	0.703

UBS: Basic Health Unit.

* Some variables did not total n = 1251 due to missing data.

** Minimum wage (2020): U$ 183,16.

- there is no p value.

The bivariate analysis between the test result and the independent variables showed an association between a positive result and age, skin color, educational level, adoption of social isolation, a positive result among household members and the presence of signs and symptoms. Regarding signs and symptoms, an association was observed between positive cases and tiredness, sore throat, anosmia, and taste disorders (Table 2).

**Table 2:** Bivariate analysis between the sociodemographic profile of the participants and the result of the serological test performed in the UBS and households, Jundiaí, 2020.

Variables		Test result		p-value
		Negative (n = 2009)	Positive (n = 422)	
		n (%)	n (%)	
Age	18 to 39 years	737 (30.4)	220 (9.1)	**<0.001**
	40 to 59 years	784 (32.3)	154 (6.4)	
	60 years or more	482 (19.9)	48 (2.0)	
Sex	Female	1,294 (53.2)	261 (10.7)	0.319
	Male	715 (29.4)	161 (6.6)	
Skin color	Black	110 (4.6)	32 (1.3)	**0.001**
	Mixed-race	427 (17.9)	113 (4.7)	
	White	1,425 (59.9)	273 (11.5)	
Educational level	Illiterate	15 (0.6)	1 (0.04)	**0.004**
	Up to elementary school	359 (14.9)	79 (3.3)	
	Up to high school	807 (33.4)	200 (8.3)	
	Complete higher education/postgraduation	819 (33.9)	135 (5.6)	
Income (in minimum wages)	No income or up to 1	135 (6.2)	27 (1.2)	0.045
	1 to 3	694 (31.6)	176 (8.0)	
	> 3	976 (44.5)	186 (8.5)	
Pandemic affected routine	Did not leave home or only for emergencies	1,274 (52.4)	243 (10.0)	0.079
	Left home to go to work	675 (27.8)	164 (6.7)	
	Did not change routine	60 (2.5)	15 (0.6)	
Preventive measures	Adhered	1,707 (70.2)	346 (14.2)	0.125
	Did not adhere	302 (12.4)	76 (3.1)	
Social distancing	Adhered	1,468 (60.4)	260 (10.7)	**<0.001**
	Did not adhere	540 (22.2)	162 (6.7)	
Familiar members testing positive	Yes	292 (12.0)	158 (6.5)	**<0.001**
	No	1,717 (70.6)	264 (10.9)	
Presence of signs or symptoms at the time of testing	Yes	279 (11.5)	160 (6.6)	**<0.001**
	No	1,730 (71.2)	262 (10.8)	
Fever	Yes	46 (10.2)	36 (8.0)	0.086
	No	244 (54.1)	125 (27.7)	
Cough	Yes	142 (31.5)	89 (19.7)	0.199
	No	148 (32.8)	72 (16.0)	
Runny nose	Yes	81 (18.0)	34 (7.5)	0.112
	No	209 (46.3)	127 (28.2)	
Headache	Yes	109 (24.1)	63 (13.9)	0.726
	No	182 (40.3)	98 (21.7)	
Body aches	Yes	89 (19.7)	51 (11.3)	0.828
	No	201 (44.6)	110 (24.4)	
Tiredness	Yes	54 (12.0)	44 (9.8)	**0.032**
	No	236 (52.3)	117 (25.9)	
Diarrhea	Yes	33 (7.3)	19 (4.2)	0.893
	No	257 (57.0)	142 (31.5)	
Sore throat	Yes	67 (14.9)	22 (4.9)	**0.016**
	No	223 (49.4)	139 (30.8)	
Shortness of breath/dyspnea	Yes	88 (19.5)	36 (8.0)	0.069
	No	202 (44.8)	124 (27.7)	
Anosmia	Yes	48 (10.6)	65 (14.4)	**<0.001**
	No	242 (53.7)	96 (21.3)	
Taste disorders	Yes	45 (10.0)	65 (14.4)	**<0.001**
	No	245 (54.3)	96 (21.3)	
Skin alterations	Yes	7 (1.6)	7 (1.6)	0.268
	No	283 (62.7)	154 (34.1)	

The final model obtained by adjusted logistic regression showed that participants with elementary and high school, as well as younger adults, had a higher risk to test positive for SARS-CoV-2. Despite significance in the behavioral block, adherence to social isolation was no longer significant in the final model. Regarding the type of sign and symptom, association was observed for anosmia and an inverse association for shortness of breath/dyspnea (Table 3).

**Table 3:** Logistic regression of sociodemographic factors, behavioral factors, signs, and symptoms of the participant undergoing serological testing in the UBS and households, Jundiaí, 2020.

Variables	Model 1			Model 2			Model 3			Final model		
	OR	95% CI	p	OR	95% CI	p	OR	95% CI	p	OR	95% CI	p
**1—Sociodemographic**												
Skin color												
Black	1.17	0.74–1.86	0.496	.	.	.	.	.	.	.	.	.
Mixed-race	1.06	0.813–1.39	0.651	.	.	.	.	.	.	.	.	.
White	1.00	.	.	.	.	.	.	.	.	.	.	.
Age (years)												
18 to 39	3.53	2.41–5.17	<0.001	3.52	2.45–5.06	<0.001	1.76	0.95–3.26	0.071	1.44	0.77–2.69	0.251
40 to 59	2.12	1.46–3.08	<0.001	2.23	1.56–3.19	<0.001	1.90	1.02–3.51	0.042	1.63	0.87–3.07	0.130
60 or more	1.00	.	.	1.00	.	.	1.00	.	.	1.00	.	.
Income*												
Up to 1	1.09	0.68–1.76	0.713	.	.	.	.	.	.	.	.	.
1 to 3	1.26	0.79–1.99	0.333	.	.	.	.	.	.	.	.	.
> 3	1.00	.	.	.	.	.	.	.	.	.	.	.
Educational level												
Elementary school	1.82	1.25–2.65	0.002	1.98	1.43–2.75	<0.001	2.70	1.62–4.52	<0.001	2.68	1.59–4.54	<0.001
High school	1.39	1.06–1.82	0.019	1.49	1.17–1.89	0.001	1.86	1.29–2.67	0.001	1.76	1.22–2.54	0.002
Higher education	1.00	.	.	1.00	.	.	1.00	.	.	1.00	.	.
**2 –Test location**												
UBS	9.16	6.84–12.25	<0.001	.	.	.	.	.	.	3.76	2.08–6.82	<0.001
Household	1.00	.	.	.	.	.	.	.	.	1.00	.	.
**3—Behavior**												
Social distancing												
Yes	1.61	1.28–2.02	<0.001	.	.	.	1.15	0.82–1.63	0.415	.	.	.
No	1.00	.	.	.	.	.	1.00	.	.	.	.	.
Familiar testing positive												
Yes	3.43	2.72–4.34	<0.001	.	.	.	1.90	1.33–2.71	<0.001	1.67	1.16–2.39	0.005
No	1.00	.	.	.	.	.	1.00	.	.	1.00	.	.
**4 –Signs and symptoms**												
Anosmia												
Yes	1.80	1.26–2.59	0.001	.	.	.	4.34	3.02–6.23	<0.001	3.68	2.56–5.28	<0.001
No	1.00	.	.	.	.	.	1.00	.	.	1.00	.	.
Taste disorder												
Yes	1.29	0.91–1.85	0.154	.	.	.	.	.	.	.	.	.
No	1.00	.	.	.	.	.	.	.	.	.	.	.
Dyspnea												
Yes	0.73	0.58–0.91	0.006	.	.	.	0.76	0.53–1.10	0.152	.	.	.
No	1.00	.	.	.	.	.	1.00	.	.	.	.	.

* Income reported as minimum wage in 2020: R$ 1,045.00.

Model 1: Skin color, age, income, and educational level.

Model 2: Age and educational level.

Model 3: Age, educational level, social distancing, household member testing positive, anosmia, and dyspnea.

Final model: Age, educational level, test location, household member testing positive, and anosmia.

## Discussion

The prevalence of positive cases was higher among participants who sought the UBS than among participants from the selected households. The factors associated with seroprevalence (positive result) included education up to elementary school compared to higher education, a household member testing positive, anosmia (loss of smell), and seeking a UBS with symptoms. The present data demonstrate the importance of extensive testing, screening the population, not only consider the symptomatic who looking for health service, but also among asymptomatic, with subclinical or mild condition. Also, is important to verify associated factors that may help to identify the most prevalent group with the COVID-19 infection.

In Brazil, the spread of COVID-19 began in large capitals, where the SARS-CoV-2 virus spread from central regions to peripheral neighborhoods. Then, the displacement of the infected population between metropolises and into the interior of the states, including due to the health infrastructure, contributed to the internalization of the virus and the involvement of all strata of the population [5,24]. This internalization was identified in Jundiai, which showed an increase in the number of cases, reaching its peak in June 2020 and a heterogeneous distribution of cases, with a concentration in peripheral regions [25].

The seroprevalence identified in the present study showed that the presence of antibodies against SARS-CoV-2 was higher among the group in UBS because they were symptomatic individuals compared to individuals who were asymptomatic, in a probabilistic sample design, justifying the association with participants who sought care at the health services (UBS). These results allow us to observe that the prevalence among individuals who present signs and symptoms of the COVID-19 is higher compared to individuals who present no or very mild symptoms [26].

Among the households group it was evidenced that the presence of antibodies against SARS-CoV-2 corroborated with what was found in a serological survey carried out at national level [27] which observed, in both stages carried out, that seroprevalence increased from 1.9% to 3.1%. Comparing with the Northeast State (Maranhão) that found the prevalence of antibodies against SARS-CoV-2 of 40, 4% in the first phase of the study and 38.1% in its second phase, the present study results are lower [28]. A literature review carried out in 2020 also noted the presence of antibodies against SARS-CoV-2 among asymptomatic individuals in Iceland (43%) and Italy (41,1%) [29]. Despite the difference in methods between the studies, these data emphasize the importance of investigating the seroprevalence between the asymptomatic, because they may present a relevant percentage in the population and, may transmit the disease due the lack to diagnosis by the health services.

It is worth mentioning that, considering a population of 418,000 inhabitants in Jundiaí/SP, there were 9,008 seropositive cases, i.e., approximately three times the number of notifications to the Epidemiological Surveillance during the study period (2,951 cases). This difference between seroprevalence and notifications has also been identified in Rio Grande do Sul by the EPICOVID project, in which the number of people in whom antibodies were detected was 6 times greater than that of notified cases [30]. Within this context, population-based studies allow to estimate the real extent of infection and also demonstrate the importance of population testing, especially for identifying asymptomatic conditions or individuals with subclinical symptoms.

In the present study, the age profile found in the population consisted of an economically active group, and this result is similar to that found in the national seroepidemiological survey conducted by the University of Pelotas [27], with a higher prevalence among adults aged 20 to 59 years, however, was different from the profile found in a Brazilian study using national data [31] which identified a higher prevalence of positive cases among men with a mean age of 59 years. This difference may be due to the design of the study that used data of notified cases, which may correspond to the profile of more severe patients, excluding mild cases of COVID-19. This fact highlights the importance of population studies since younger people are considered an important group for contamination and transmission of the disease.

Another important sociodemographic aspect found was the higher seroprevalence was found among subjects with lower education, corroborating a study carried out in the United States, which, in addition to years of schooling, that study also reported an association with black skin color [32]. It is noteworthy that the present study also identified, in the bivariate analysis, black skin color as an associated factor; however, this variable lost significance after adjusting for the other factors in the logistic regression. This information indicates that, although COVID-19 affects all strata of the population, aspects of inequity must be highlighted with can also be related to people who have difficulty in social distancing and who need to leave home to go to work are more exposed. This reality also points to an important fact which deserve attention for health policies, because is described the current economic crisis that has been the largest since the Second World War, with millions of unemployed individuals and people in poverty [33]. In this way, public policies are needed to expand the opportunities of those who were economically more affected by the pandemic, considering that these individuals are also more exposed to infection and other diseases and put the population at risk [33].

In the present study, the prevalence of seropositivity was higher among participants with a familiar contact that tested positive. According to a study carried out in households in China, the household transmission rate was 16.3%, with age and spousal relationship being risk factors [34]. Contact with an infected individual increases the risk of infection and social distancing is therefore an important preventive measure [10–12]. Quarantine of positive patients since the onset of symptoms is effective in preventing transmission and the occurrence of new cases.

Among the participants who tested positive, the anosmia was one of the associated factors identified in the present study, even, after adjusting the logistic regression models. However, anosmia appeared as a protective factor for the most severe forms of infection. The report of shortness of breath/dyspnea was identified as a protective factor, however, after adjusting the model with other regression factors, there was no significance in the present study. Other study carried out in primary care services in the region of Tarragona [35] identified anosmia as a protective factor for the critical outcome of the infection and dyspnea was associated with the risk of developing more severe forms of the disease, the inverse association we have found in the present study, probably this difference could be attribute to disease levels, mild and severe [35]. It is noteworthy that, from the present study, it was not possible to identify which variant of SARS-CoV-2 was circulating to be associated with the symptom of anosmia.

The behavior variables studied as social distance and use of masks were associated in bivariate analyses but didn’t remain after adjusted, considering the importance of sociodemographic factors associated with the COVID-19 in the present study. However, it is important to emphasize that these measures are essential to contain the pandemic [7].

This study was the result of cooperation between a teaching institution and the city hall, reinforcing the idea that the integration of scientific knowledge, health services and health management is necessary to achieve good public health outcomes in SUS [36]. Effective testing strategies are very important to efficiently utilize the tests and resources provided by the government for the detection and control of the coronavirus, in addition to providing results for a poorly studied population.

One limitation of this seroepidemiological survey was the difficult of training the teams for data collection and application of the tests in the pandemic context. The training of the volunteers in the application of the questionnaire and use of the app was a way to overcome this barrier. Other limitation was the different study design for sample selection in both groups, that contribute with the greater positivity in symptomatic individuals who seek the health service. Other important point to highlight is the sensitivity and specificity of the rapid test, that depends on the period of the onset symptoms, and it is not clear if the antibody tests are able to detect lower antibody levels likely seen with milder and asymptomatic COVID‐19 disease, which represents a challenge to screening the population [26].

Besides the limitation found, screening COVID-19 infection it was very important to plan measures of mitigation the pandemic, and rapid tests are easy and fast to apply and to obtain the result, making possible in populational studies to comprehend the extension of the infection in the location. However, the results should be interpreted carefully based on diagnostic method chosen.

This study contributed to the understanding of the seroprevalence of SARS-CoV-2, that was higher among symptomatic and asymptomatic individuals and to the identification of the epidemiological profile of the disease, as well as health behaviors related to the pandemic. Sociodemographic factors were associated with being positive for antibodies, and anosmia and having familiar who tested positive. Extensive COVID-19 testing is recommended as a preventive strategy. We highlight the importance of studies such as the present one that are representative of the population and that investigate factors associated with infection so that more accurate and comprehensive strategies can be implemented.

## Supporting information

S1 Data (SAV)
